# Serum Inflammatory Markers and Their Associations with the Integrity of the Cingulum Bundle in Schizophrenia, from Prodromal Stages to Chronic Psychosis

**DOI:** 10.3390/jcm11216352

**Published:** 2022-10-27

**Authors:** Anna Michalczyk, Ernest Tyburski, Piotr Podwalski, Katarzyna Waszczuk, Krzysztof Rudkowski, Jolanta Kucharska-Mazur, Monika Mak, Katarzyna Rek-Owodziń, Piotr Plichta, Maksymilian Bielecki, Wojciech Andrusewicz, Elżbieta Cecerska-Heryć, Agnieszka Samochowiec, Błażej Misiak, Leszek Sagan, Jerzy Samochowiec

**Affiliations:** 1 Department of Psychiatry, Pomeranian Medical University, 71-460 Szczecin, Poland; 2 Department of Health Psychology, Pomeranian Medical University, 71-460 Szczecin, Poland; 3 Department of Neurosurgery, Pomeranian Medical University, 71-252 Szczecin, Poland; 4 Department of Laboratory Medicine, Pomeranian Medical University, 70-111 Szczecin, Poland; 5 Department of Clinical Psychology, University of Szczecin, 71-017 Szczecin, Poland; 6 Department of Psychiatry, Division of Consultation Psychiatry and Neuroscience, Wroclaw Medical University, 50-368 Wroclaw, Poland

**Keywords:** schizophrenia, first episode psychosis, UHR, diffusion tensor imaging, cingulum bundle, inflammatory markers, cytokines

## Abstract

Peripheral cytokines may affect the brain through chronic activation of microglia and, as a result, can potentially lead to decreased integrity of white matter of cingulum bundle (CB). Therefore, the aim of the study was to analyze the relationships between peripheral inflammatory markers and the integrity of the CB in various states: from healthy controls, through prodromal states and first-episode psychosis, to long-term schizophrenia. The integrity of the CB was measured using diffusion tensor imaging. We analyzed six parameters: CRP, IL-6, IL-8, IL-10, TNF-α, and IFN-γ. We found that levels of IL-6 and IFN-γ differed significantly between groups. Initial analysis showed some correlations between the inflammatory markers and CB integrity, in particular a correlation with IL-6 that was present in several groups. However, none of the analyzed parameters were associated with the integrity of the CB after correction for multiple comparisons. Conclusions: Our results supported our hypothesis that there are increased levels of inflammatory markers in psychotic disorders, but did not allow to confirm our hypothesis that there is a link between increased peripheral inflammatory markers and decreased integrity of the CB. However, we found some interesting trend levels that need to be verified in larger studies.

## 1. Introduction

Schizophrenia affects about 0.5–1% of the general population [1]. The course of the disease is varied and difficult; however, early diagnosis and treatment may improve outcomes [2]. Usually, attenuated and transient psychotic symptoms appear several years before the first episode of full-blown psychosis (FEP), and their presence is associated with a high risk of developing psychosis. This condition is known as “ultra-high risk” (UHR), “clinically high risk”, an “at-risk mental state”, or a “prodromal state” [3]. Regardless of the name, early recognition of patients with UHR enables appropriate preventive strategies to be implemented. It has been shown that the duration of untreated psychosis is the most critical prognostic factor in schizophrenia [4]. Diagnosis and prognosis in UHR patients is currently based mainly on psychiatric examination and medical history, but objective prognostic markers based on the biological background of the disease are being sought. However, despite many years of research, the biological basis of schizophrenia is still poorly understood.

Chronic subclinical inflammation is considered to be a substantial factor in the development of schizophrenia. Meta-analyses show altered levels of peripheral inflammatory markers in both people at high risk of developing psychosis [5,6,7] and in FEP patients [6,8,9] or those with chronic psychosis [10,11,12]. Peripheral cytokines may affect the brain through the activation of microglia. Microglia, the immune cells of the central nervous system (CNS), are usually in a quiescent state and support proper brain development and functioning by controlling cell apoptosis and influencing neural circuits. Temporarily increased microglial activity protects the CNS during infections and when tissue is damaged. However, prolonged continuous activation may disrupt physiological functions, stimulate apoptosis of oligodendrocyte precursor cells, and lead to structural changes in white matter [13,14,15].

Structural abnormalities of the white matter in various brain regions have been reported not only in chronic schizophrenia, but also in its early and prodromal stages [16]. According to the literature, diffusion tensor imaging (DTI; alone or in combination with other neuroimaging techniques) can reliably distinguish between UHR, FEP, or SCH patients and HC, with classification accuracies over 65%, 84%, and 98%, respectively [17,18,19]. One of the regions of particular interest is the cingulum bundle (CB)—the white matter tract that links frontal, parietal, and medial temporal sites and connects subcortical nuclei to the cingulate gyrus [20]. Meta-analyses show reduced white matter integrity of the CB—reduced fractional anisotropy (FA) and/or increased mean diffusivity (MD)—in patients with FEP and SCH [21,22,23]. Similar abnormalities are observed in UHR [24,25]. Our previous study also showed reduced integrity of the CB in schizophrenia patients compared to controls and its possible association with impairments in executive functions [26].

Animal studies have shown that prenatal exposure to inflammation induces changes in tract fractional anisotropy throughout the fronto-striatal-limbic circuits [27]. In humans, one study on very preterm infants showed that neonatal sepsis was associated with reduced integrity of the cingulum bundle [28]. There are also individual publications indicating correlations between serum concentrations of some inflammatory markers and abnormalities in the white matter microstructure of various brain regions, both in the general population [29] and in patients with schizophrenia [30,31] or first-episode psychosis [32]. For example, our previous study showed correlations between reduced integrity of the corpus callosum and increased levels of IFN-γ in SCH and IL-6 in HC [33]. Recently, Thomas et al. [34] showed that systemic inflammation is associated with reduced white matter integrity of the CB in patients with depression. Correlations between the integrity of various brain regions, including the CB, and levels of peripheral inflammatory cytokines were also observed in patients with bipolar disorder [35]. However, as far as we are aware, there are no studies that investigate associations between inflammatory markers and integrity of the CB in schizophrenia and its prodromal stages.

Therefore, the main aim of this study was the analysis of relationships between the parameters of inflammation in the peripheral blood and the integrity of the white matter of the cingulum bundle in various states: from healthy people, through prodromal states and first episode psychosis, to long-term schizophrenia. We hypothesize that psychotic disorders are associated with changes in levels of inflammatory markers and that these changes are correlated with reduced fractional anisotropy and/or increased mean diffusivity of the CB.

## 2. Materials and Methods

### 2.1. Participants and Procedure

This study was performed with four independent groups differing in health status: healthy controls with no psychotic disorders (HC), patients with ultra-high risk of psychosis (UHR), patients with first-episode psychosis (FEP), and patients with chronic schizophrenia (SCH).

The HC group consisted of 34 healthy volunteers aged 22–48 years old recruited through information spread by students and workers from the local universities; among the inclusion criteria were having no mental or neurological disorders; this was verified by psychiatric evaluation and a structured self-report questionnaire. The UHR group consisted of 16 patients aged 18–32 years old who met the criteria for ultra-high risk of psychosis set out in the Polish translation of the Structured Interview for Prodromal Syndromes (SIPS; [36,37]), recruited from the Mental Health Clinic at the Clinic of Psychiatry at the Pomeranian Medical University (PMU) in Szczecin, Poland. The FEP group consisted of 32 patients aged 19–42 years old with the presence of previously untreated psychotic symptoms with recent onset and no remission since the beginning of symptoms, who had been diagnosed with a schizophrenia spectrum disorder (F20–F29) based on the International Classification of Diseases and Related Health Problems (ICD-10; [38,39]). Patients were recruited from inpatients and outpatients at the Clinic of Psychiatry at the PMU. The SCH group consisted of 71 patients in stable mental state (none of the patients were in an acute state of psychosis outbreak on the basis of psychiatric evaluation) aged 25–57 years old, recruited from inpatients, day treatment patients, and outpatients at the Clinic of Psychiatry at the PMU. Only patients who had been diagnosed with schizophrenia for at least ten years were enrolled. The diagnosis was confirmed prior to the start of the study by a properly licensed psychiatrist based on a structured clinical interview in line with the ICD-10 and the Mini-International Neuropsychiatric Interview (MINI; [40]).

Ability to comprehend the test procedures and undergo a neuroimaging procedure as well as a stable physical state were inclusion criteria common to all study groups, including controls. Exclusion criteria in all groups were: concomitant psychiatric or neurological disorders based on the ICD-10 (with the exception of the disorder that was the basis for inclusion in a given group), evident coincidence of symptoms with use of psychoactive substances, a history of cerebral or cranial injury, current inflammatory diseases (i.e., clinical symptoms of current infections; past infections and autoimmune diseases were not taken into account in the study), and severe somatic conditions (i.e., cancer).

Study participants underwent psychiatric evaluation, blood sampling, and a DTI procedure. All psychiatric examinations were performed by members of the research team who were licensed and properly trained psychiatrists. Chlorpromazine equivalent was calculated on the basis of defined daily doses [41]. The severity of psychopathological symptoms was assessed using SIPS in UHR and the Positive and Negative Syndrome Scale (PANSS; [42]) taking into account five psychopathological dimensions [43,44] in FEP and SCH. In the UHR and FEP groups, the study procedures were performed a maximum four weeks after admission due to lack of compliance in the acute phase of disease. In all groups, blood sampling and the DTI procedure were performed on the same week—mostly on the same day.

The study was approved by the Bioethical Commission of the Pomeranian Medical University in Szczecin (study no KB-0012/49/17/A-1 and KB-0012/159/17/A-1). All participants gave informed written consent to participate in the study.

### 2.2. Acquisition of DTI Data and Image Processing

DTI was performed using a 3.0 Tesla scanner (General Electric Signa HDxt). A single shot pulse sequence with diffusion-weighted echo planar acquisition was used to obtain DTI data with the following imaging parameters: repetition time—11.675 s; echo time—82.80 ms, number of excitations—2; acquisition time—10 min, 19 s; matrix—96 × 96; field of view—240 × 240 mm; slice thickness—3 mm; slice gap—0.50; b value—1000 s/mm^2^; and 25 diffusion gradient directions.

**Figure 1:**
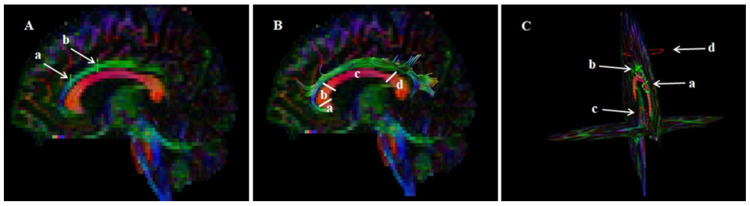
Diffusion tensor imaging tractography of the cingulum bundle with fractional anisotropy color maps. Green, red, and blue colors represent fibers running along the axis (anterior–posterior, left–right, and superior–inferior, respectively). (**A**) sagittal slice with the location of two regions of interest (ROIs; a—ROI1, b—ROI2); (**B**) coronal view with final location of ROIs (a—ROI1, b—ROI2, c—ROInot1, d—ROInot2); (**C**) sagittal slice with reconstructed cingulum bundle (arrow) and sagittal section of corpus callosum (a—rostrum, b—genu, c—body, d—splenium). Reproduced from [26] with permission from Elsevier.

The main analysis was preceded by preprocessing, quality control, and fiber tract visualization with the ExploreDTI tool [45]. For this step, DICOM files were converted to the *.nii format. Images were checked to make sure their sides matched the originals and then corrected for signal drift, eddy current, and effects due to motion. Artifacts were removed. After visual inspection of data quality, they served as the basis for whole-brain tractography using a constrained spherical deconvolution tracking algorithm. Visualization of CB was based on an FA color map in the coronal plane. Two regions of interest (ROIs) were used in analysis: ROI1 at the height of the posterior margin of the genu of the corpus callosum and ROI2 on the level of foramina of Monro on the coronal plane along the CB. The parts of the tracts that are not anatomically involved in the CB were excluded (as ROInot; Figure 1). Finally, the ExploreDTI Descriptive Statistic function was used to automatically calculate the fractional anisotropy and mean diffusivity of the fiber tract.

### 2.3. Inflammatory Markers

Serum samples were obtained from blood collected in the fasting state from a peripheral vein between 7 and 9 in the morning; they were centrifuged (1000× *g*, 10 min, 20 °C) after clot formation within an hour of blood donation. Serum was transferred to new tubes and immediately frozen and stored at −80 °C until the assays were performed. The time of samples storage was similar in all groups and the determination of the levels of inflammatory markers was performed at the same time for all groups, i.e., measures were performed after collecting the appropriate number of samples per plate, regardless of their belonging to a given group and the recruitment was conducted simultaneously for all groups. To ensure high sensitivity, levels of inflammatory markers were measured with ELISA kits, using high sensitivity assays or a chemiluminescent detection system. According to the manufacturers, the sensitivities of the kits were: 0.35 pg/mL for IL-6 (Human IL-6 QuantiGlo ELISA Kit; R&D Systems, Inc., Minneapolis, MN, USA), 0.97 pg/mL for interleukin-8 (IL-8; Human IL-8 QuantiGlo ELISA Kit; R&D), 0.481 pg/mL for TNF-α (Human TNF-α QuantiGlo ELISA Kit; R&D), 0.05 pg/mL for interleukin-10 (IL-10; IL-10 ELISA Kit, HS; Invitrogen, Carlsbad, CA, USA), 0.69 pg/mL for interferon-gamma (IFN-γ; Human IFN-γ HS ELISA Kit; Abcam, Cambridge, UK), and 0.1 mg/L for C-reactive protein (CRP; CRP HS ELISA; DRG Instruments GmbH, Marburg, Germany). Assays were performed according to the manufacturer’s protocols. A SYNERGY HTX multi-mode reader (BioTek Instruments Inc., Winooski, VT, USA) was used to measure absorbance/luminescence in the samples and a Gen5 Microplate Reader and Imaging Software (BioTek) were used to generate best fit standard curves.

### 2.4. Statistical Analysis

**Figure 2:**
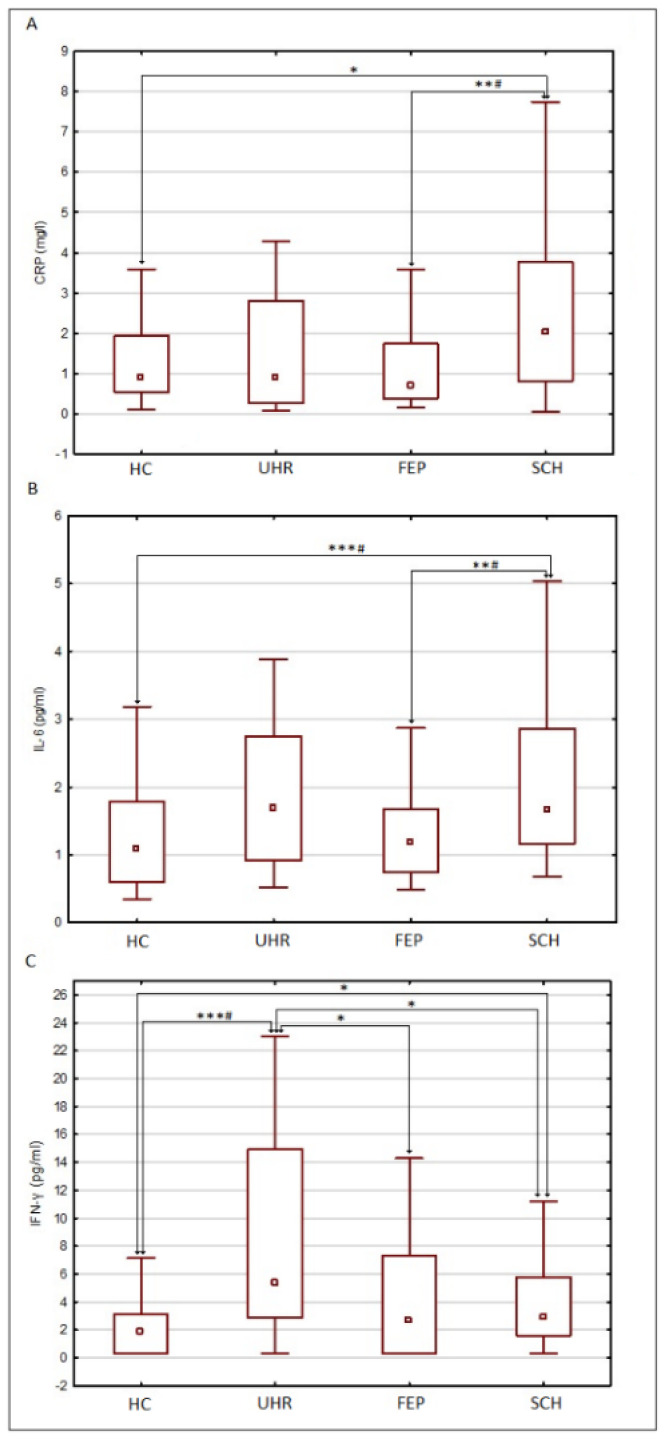
Comparison between groups of the serum concentrations of (**A**) CRP, (**B**) IL-6, and (**C**) IFN-γ. HC-healthy controls, UHR-patients with ultra-high risk of developing psychosis, FEP-patients with first episode psychosis, SCH-patients with chronic schizophrenia, CRP—C-reactive protein, IL-6—interleukin-6, IFN-γ—interferon-γ. Data presented as median (point), interquartile range (box), and non-outlier range (whiskers), * *p* < 0.05, ** *p* < 0.01, *** *p* < 0.001 (without correction for multiple comparisons); # significant differences after correction for multiple comparisons.

Statistical analysis was performed using Statistica 13.3 software (TIBCO Inc., Palo Alto, CA, USA)). The Shapiro–Wilk test was used to verify the normality of distributions. Most of the analyzed variables, especially concentrations of inflammatory markers, were non-normally distributed, nor were the log-transformed values; therefore, non-parametric tests were used. For qualitative variables, group comparisons were performed using a chi-square test. For quantitative/ordinal variables, group comparisons were performed using the Mann–Whitney U test for two groups and Kruskal–Wallis H test for more than two groups. Correlations were analyzed using Spearman’s rank correlation coefficient (ρ). In all analyses, *p* < 0.05 was taken as the threshold of statistical significance. Bonferroni correction was used in some analyses to verify the significance of differences/correlations across multiple comparisons. In Table 1, the *p* value was corrected for the number of analyzed parameters (*n* = 21), in Table 2 and Table 3 it was corrected for the number of analyzed inflammatory markers (*n* = 6) and in Figure 2 it was corrected for the number of comparisons between groups within a given inflammatory marker (*n* = 6).

Analysis of the tests’ powers performed for correlation analyzes showed that the necessary minimum group size for detection of moderate correlations (ρ > 0.50) between the levels of inflammatory markers and CB integrity with a power of >0.80 was 29. This assumption was met for the SCH and HC groups. The UHR and FEP groups were smaller due to problems with recruitment and failure to complete the DTI examination by some patients (the correlation analyzes included 12 patients with UHR and 19 with FEP), but they were sufficient for detection of highly correlated (ρ > 0.70) measures with the desired power (0.80).

## 3. Results

### 3.1. Group Comparisons

The analyzed groups of patients and controls did not significantly differ in terms of sex or years of education, but they did differ in terms of age, BMI, and smoking status (Table 1). Additionally, the patient groups differed in duration of illness, number of exacerbations, and type and doses of antipsychotic medications taken (Table 1).

Among inflammatory markers, there were significant differences in serum CRP, IL-6, and IFN-γ levels (Table 1). Differences in IL-6 and IFN-γ remained significant after correction for multiple comparisons. Details about direct group comparisons are presented in Figure 2.

Despite log-transformation, most of the analyzed variables were not normally distributed, therefore we were not able to perform any multivariate tests taking into account age, BMI, and/or smoking status as covariates. Instead, we analyzed associations between these potential confounding factors and levels of inflammatory markers in all analyzed groups.

We found significant positive correlation between age and IL-6 in HP and negative between age and TNF-α in UHR. BMI was positively correlated with CRP in HP and UHR and with IL-6 in HP. There were no significant differences in IL-6, IL-8, TNF-α, and IFN-γ between smokers and non-smokers in the SCH or FEP groups, but in the SCH group, smokers had somewhat lower CRP and IL-10 levels.

Among the patient groups, duration of illness correlated negatively with TNF-α in SCH and positively with TNF-α and IL-10 in UHR. Number of exacerbations correlated negatively with IL-8 in SCH. Antipsychotic medications were associated with TNF-α levels in FEP. Antipsychotic doses, presented as chlorpromazine equivalent, correlated positively with IL-6 in SCH participants and IFN-γ in UHR participants.

We have also found some correlations between inflammatory markers and GAF and psychopathological symptoms. The score in GAF was negatively correlated with IL-10 in UHR. The severity of positive symptoms in PANSS was negatively correlated with IFN-γ in FEP and positively correlated with IL-6 in SCH. The severity of depressive symptoms in PANSS was negatively correlated with IL-8 in SCH. The severity of positive symptoms in SIPS was negatively correlated with IL-8 in UHR. Complete results of analysis of associations between inflammatory markers and demographic, anthropometric and clinical parameters, and psychopathological symptoms are presented in Supplementary Table S1.

### 3.2. Correlations between Inflammatory Markers and the Integrity of the Cingulum Bundle

Basic analysis of correlations between inflammatory markers and the integrity of the CB showed negative correlations between IL-6 and FA of the left CB in UHR and between IFN-γ and FA of the left and right CB in HC (Table 2). Taking into account values of MD, there was a positive correlation between IL-6 and the MD of the left CB in SCH and negative correlations between IL-8 and the left and right CB in UHR and left CB in HC (Table 3). However, none of the analyzed correlations reached statistical significance after correction for multiple comparisons.

## 4. Discussion

Among the analyzed inflammatory markers, CRP, IL-6, and IFN-γ showed significant differences between groups, but only differences for IL-6 and IFN-γ remained significant after correcting for multiple comparisons. The most differences were found between the HC and SCH groups. SCH was associated with higher levels of CRP, IL-6, and IFN-γ. However, due to the observed positive correlation between age and/or BMI and IL-6 and CRP levels in HC, we cannot exclude the possibility that these factors may have a confounding effect on the results of the analysis. SCH and HC did not differ significantly in age, but a significantly higher BMI was observed in SCH. The non-normal distribution of variances, even after their log transformation, prevented us from performing a multifactorial analysis. However, our previous study used some of the same groups of patients and controls; it found that, in multiple binary logistic regression, taking into account age, BMI and IL-6, participants with higher IL-6 had greater odds of having schizophrenia (all else being equal; [33]). Our results are in line with meta-analytical data, which consistently shows increased levels of CRP [12,46,47] and IL-6 in schizophrenia [8,48]. The available data for IFN-γ are contradictory. According to the meta-analysis of Miller et al. [8], IFN-γ is increased in acute-relapsed chronic schizophrenia patients and does not change after treatment, but according to Goldsmith et al. [48], only acute relapsed patients have increased IFN-γ, while in stable patients the level of IFN-γ is decreased. These differences may result in part from differing characteristics among the study participants, such as disease duration or treatment strategy; further investigation is needed.

The UHR group showed significantly higher levels of IFN-γ compared to all other groups, including HC. It should also be noted that there was a relatively large variation in IFN-γ levels within this group. According to the meta-analyses of Park et al. [7] and Misiak et al. [5], IFN-γ is not altered in UHR patients compared to controls; however, these analyses contain the results of only 2 and 6 studies that measured this parameter, respectively. Our results are very similar to the study of Kelsven et al. [49], which also obtained high mean concentration and high standard deviation for IFN-γ in this group of patients (mean 13.85, SD 22.26). We did not find any correlations between IFN-γ and any demographic or anthropometric parameters, so they should not affect the results of the analysis. However, we found a positive correlation between the levels of IFN-γ and antipsychotic dose in UHR. This group of patients varied greatly in terms of antipsychotic treatment, which may explain the large variation in IFN levels. We did not find any associations for other inflammatory markers, but we observed a statistical trend for higher levels of IL-6 in UHR compared to HC (*p* = 0.07). Increased levels of IL-6 in UHR patients compared to controls were also found in meta-analyses [5,7]. The small sample size and significantly higher age of HC compared to UHR could be the reason for not reaching statistical significance for IL-6 in our study. Another explanation could be the use of antidepressants by nearly a third of patients in UHR group. In this group, lower levels of IL-6 and IL-10 were observed in patients taking antidepressants compared to patients not taking this type of drug (statistical trend, *p* < 0.10). This observation is consistent with the literature data that show that antidepressive treatment reduces levels of these inflammatory markers in patients with major depressive disorder [50]. In FEP, we did not find significant differences in inflammatory markers compared to HC. Meta-analyses often show increased levels of IL-6 and TNF-α in FEP compared to controls [51]. The lack of these links in our study may be partially due to the younger age of FEP compared to HC. Additionally, in our study, procedures were performed with a delay of up to four weeks from admission to hospital due to the common lack of compliance in the acute phase of disease, and almost all patients were taking antipsychotics. Some meta-analyses have suggested that antipsychotic drugs may reduce plasma levels of IL-6 [52,53,54] and TNF-α [54].

It is worth noting that, as shown in the Supplementary Table S1, the levels of inflammatory markers may be associated with modifiable factors such as smoking or BMI, may be affected by disease duration, type and doses of antipsychotic drugs, and may be related to the severity of psychopathological symptoms. These associations may play an important role in the context of the complexity of the analysis of inflammatory markers in schizophrenia and require further investigation.

Analysis of correlations between serum inflammatory markers and the integrity of the CB did not provide strong evidence supporting the thesis about the relationship between peripheral inflammation and CB microstructure disruptions. Several correlations were observed; however, their level of significance was usually close to the threshold, and none of them remained significant after correction for multiple comparisons. Some correlations were observed only in one of the analyzed groups and for only one of the analyzed markers of disruptions of CB integrity (FA/MD), which suggests that they might be mere coincidence. A correlation between IL-8 and MD of the left/right CB appeared in two groups—HC and UHR—but the direction of this association was opposite to what was expected and suggests that increased levels of this proinflammatory cytokine are associated with increased integrity of CB (reduced MD). We found no studies that support such a relationship. Another relationship that was observed in more than one group was the negative correlation between IL-6 and FA of the right CB in HC, a negative correlation between IL-6 and FA of the left CB in UHR, and a positive correlation between IL-6 and MD of the left CB in SCH. All of these relationships were in the expected directions; that is to say, increased levels of IL-6 were associated with reduced integrity of the CB, understood as either reduced FA or increased MD. They were significant only for one marker of white matter integrity in the given study group (FA/MD); however, these indicators measure a different kind of microstructural changes and they do not have to match [55]. Perhaps inflammation-related changes in the brain microstructure are different in the early stages of the disease and in chronic schizophrenia. The observed correlations do not result from the relationship between serum level of IL-6 and age or BMI, as we did not find any correlation of these parameters with the CB integrity measures mentioned above. Similar associations for this cytokine were previously found in various brain regions in patients with schizophrenia [30,33]; however, neither of these studies analyzed the region of the cingulum bundle, which makes it difficult to compare our results with the data from the literature. Taking into account the likely partially common biological basis of various mental disorders, we analyzed the results of similar studies conducted on patients with other mental disorders. Thomas et al. [34] found associations between peripheral CRP levels and the integrity of the CB in patients with depression, but he did not analyze any other inflammatory markers; in contrast, correlation with CRP was not observed in our study. Benedetti et al. [35] analyzed a wider panel of inflammatory markers, including IL-6, and their associations with CB integrity in patients with bipolar disorder. They found associations between increased levels of proinflammatory cytokines TNF-α, IL-8, and IFN-γ and reduced FA and increased MD of various brain regions, including the CB. However, they did not find such a correlation for IL-6. Both our study and all those cited were performed with relatively small groups of patients, and the levels of cytokines were analyzed in a single sample. Therefore, the results reflect their instantaneous concentration at the time of collection, which may be influenced by many factors and not necessarily properly reflect their average levels over a long period of time, which could have influenced the results of the analyses and reduced the quantity of observed significant associations. Furthermore, prospective studies on larger groups are needed to verify that the observed correlations reflect a real association between these parameters. However, even assuming that these associations were not accidental and they would find confirmation on larger sample sizes, it should be noted that the observed correlations were not strong and their clinical significance is probably limited.

It should be emphasized that in our study the vast majority of participants received antipsychotics. Antipsychotic treatment is an important element that can potentially affect both the level of inflammatory markers and the integrity of white matter [8,54]. It was shown in a co-culture model that pretreatment of atypical antipsychotics suppresses oligodendrocyte damage from interferon-γ-stimulated microglia [56]. Assuming that the putative mechanism of the influence of peripheral inflammatory markers on white matter is based on microglia activation, the presence of blockers of the effect of microglial activation on oligodendrocytes may have reduced the correlations between serum inflammatory markers and the integrity of various brain regions. In our study, we found no associations between the use of specific types of antipsychotic drugs and the integrity of CB (data not shown), but this could be due to the very low numbers of people untreated with atypical antipsychotics in particular groups of patients. However, we observed a positive correlation between the equivalent of chlorpromazine and the FA for left and right CB in FEP (ρ = 0.57, *p* = 0.006 and ρ = 0.52, *p* = 0.049, respectively). We have also found positive correlations between chlorpromazine equivalent and IFN-γ in UHR and IL-6 in SCH and the association between type of antipsychotics used and TNF-α in FEP (Supplementary Table S1), which also could affect the results. In order to verify the influence of antipsychotics on our results, studies on larger numbers of patients are needed, including a group of drug-naive patients.

Given the key role of microglia in the putative mechanism of the influence of peripheral inflammatory markers on the integrity of CB, we must also take into account that peripheral cytokines are not the only activating factor for microglia. Neuroinflammation may result from many factors such as infections, autoimmune diseases, metabolic disorders and even mental stress or unhealthy lifestyle. For example, it was shown that obesity induces production of reactive oxygen species by neuronal cells, which may activate microglia and support neuroinflammation. It was also shown that unhealthy diet disrupts gut microbiome, which results in increased blood–brain barrier permeability and neuroinflammation [57]. Chronic activation of microglia stimulates apoptosis of oligodendrocyte precursor cells, and may lead to structural changes in white matter [13,14,15].

The major limitation of this study was the relatively small sizes of UHR and FEP groups with complete DTI data. The number of patients in these groups was enough to detect strong correlations with the appropriate power, but moderate correlations may have remained undetected. Therefore, the results in these groups should be treated as pilot studies. In HC and SCH, the group sizes allowed the detection of moderate correlations with adequate power, but weak correlations could go undetected. However, even if we missed some of these weaker correlations, their clinical significance would probably be small, if any. The second limitation was the cross-sectional nature of this study. We analyzed groups at different stages of the disease, but as it was not a prospective study, it did not allow for the drawing of any cause-and-effect conclusions. The third limitation was the mismatching of groups in terms of age, BMI, and smoking status. The main aim of our study was to analyze the correlations between inflammatory markers and the integrity of the CB within the groups, for which group matching is not needed; therefore, we focused on recruiting participants that were representative of a given group. It is natural that SCH patients are older than UHR/FEP patients and that patients with schizophrenia are more often overweight or obese and smoke cigarettes compared to controls [58,59]. These parameters could influence the group comparisons as described above. It is also worth noticing that in the control group the median BMI was 25, which indicates the presence of overweight in half of participants in this group. This sample seems representative for source population as according to World Health Organization [60] the prevalence of overweight among adults of >18 years old in Poland in 2016 was estimated at 62.4%, but the overweight could induce low-level inflammation in controls. The fourth limitation was that the accuracy of DTI measurements was limited by its low spatial resolution at 3T. Fifth, we based the measurements of inflammatory markers concentrations on single samples. All samples were taken on an empty stomach in the morning to ensure the greatest uniformity possible; however, many unidentified factors could have influenced the concentration of these parameters on a given day. Serial measurements on different days would probably give a better picture of the severity of chronic subclinical inflammation in participants. Finally, some factors with possible influences on levels of inflammatory markers, such as taking anti-inflammatory medications and past infections or autoimmune diseases, were not taken into account in the study. We also did not include cannabis consumption, which could influence both inflammatory process and white matter microstructure [61,62]. We plan to include these factors in further research.

## 5. Conclusions

Our study supports the existence of increased levels of inflammatory markers in psychotic disorders, including the UHR state, and shows that the profile of disrupted cytokines depends on the state of the disease. Regarding the second hypothesis about the association between peripheral inflammation and CB microstructure, the observed correlations were not strong, did not remain significant after correction for multiple comparisons, were usually present only in one group for one analyzed inflammatory markers, and did not find confirmation in another analyzed CB integrity marker (MD/FA). This indicates that the observed correlations were likely coincidental. The exceptions were correlations with IL-6, which appeared in several groups and whose direction was consistent with the assumptions; this may indicate that they play an important role and require further investigation.

## Figures and Tables

**Table 1:** Demographic, anthropometric, and clinical data for patients and controls.

Parameter	HC	UHR	FEP	SCH	H/x2
*n*	34	16	30	71	
Women	19 (56)	8 (50)	18 (60)	31 (44)	2.80
Age (years)	36 ± 8 (37)	24 ± 5 (25)	28 ± 6 (27.5)	39 ± 7 (39)	56.79 ***#
Years of education	15 ± 3 (15.5)	13 ± 3 (12)	13 ± 3 (12)	13 ± 3 (12)	6.96
BMI (kg/m^2^)	26 ± 4 (25)	23 ± 3 (22)	24 ± 4 (23)	28 ± 5 (28)	28.92 ***#
Smoking cigarettes	0 (0)	0 (0)	4 (13)	23 (32)	20.51 ***#
Duration of illness (years)	-	1.0 ± 1.2 (0.5)	0.7 ± 1.3 (0.3)	15.2 ± 5.5 (13)	84.04 ***#
Exacerbations ^	-	5.1 ± 6.4 (1.0)	1.1 ± 0.3 (1)	6 ± 4 (5)	59.68 ***#
Antipsychotic medications:					41.92 ***#
Atypical	-	7 (44)	26 (87)	49 (69)	
Atypical and typical	-	1 (6)	2 (7)	20 (28)	
Typical	-	0 (0)	1 (3)	3 (4)	
None	-	8 (50)	1 (3)	2 (3)	
Chlorpromazine equivalent (mg)	-	138 ± 210 (17)	483 ± 305 (450)	666 ± 308 (640)	43.23 ***#
Antidepressant medications	-	5 (31)	0 (0)	2 (3)	
Global functioning in GAF	-	62 ± 14 (65)	59 ± 17 (56)	56 ± 15 (60)	2.60
Psychopathological symptoms in PANSS:					
Positive	-	-	12 ± 5 (12)	8 ± 4 (6)	4.01 ***#
Negative	-	-	17 ± 7 (17.5)	17 ± 6 (16)	−0.15
Disorganization	-	-	15 ± 5 (14)	12 ± 4 (11)	2.61 **
Affect	-	-	10 ± 4 (9.5)	9 ± 3 (8)	1.75
Resistance	-	-	5 ± 2 (4)	5 ± 2 (4)	1.44
Psychopathological symptoms in SIPS:					
Positive	-	6.3 ± 4.0 (6)	-	-	
negative	-	11.5 ± 3.4 (14)	-	-	
Disorganization	-	4.5 ± 3.4 (3)	-	-	
General	-	8.0 ± 4.0 (8)	-	-	
CRP (mg/L)	2.45 ± 5.23 (0.89)	2.70 ± 4.95 (0.91)	1.24 ± 1.29 (0.71)	6.28 ± 15.89 (2.03)	10.67 *
IL-6 (pg/mL)	1.32 ± 0.84 (1.08)	1.85 ± 1.08 (1.68)	1.34 ± 0.65 (1.20)	2.76 ± 3.48 (1.65)	16.08 **#
IL-8 (pg/mL)	11.56 ± 7.29 (10.19)	9.81 ± 3.30 (8.83)	8.51 ± 3.50 (7.62)	12.43 ± 21.63 (9.69)	7.77
IL-10 (pg/mL)	1.26 ± 1.24 (1.18)	1.02 ± 0.74 (1.14)	5.15 ± 21.86 (0.82)	4.43 ± 22.14 (1.42)	5.01
TNF-α (pg/mL)	6.91 ± 2.38 (6.26)	7.18 ± 3.88 (6.26)	6.70 ± 2.51 (5.88)	6.69 ± 2.42 (6.12)	0.90
IFN-γ (pg/mL)	2.86 ± 3.96 (1.81)	11.88 ± 16.02 (5.30)	4.50 ± 5.35 (2.62)	9.21 ± 30.65 (2.94)	14.67 **#

Data are presented as either *n* (%) or mean ± SD (median) * *p* < 0.05, ** *p* < 0.01, *** *p* < 0.001 (without correction for multiple comparisons)*;* # significant differences after correction for multiple comparisons. ^ In the UHR group, exacerbations mean the number of periods with present symptoms; in FEP and SCH, conditions requiring hospitalization (in FEP, there was one patient with 2 exacerbations because of self-reporting of exacerbation with spontaneous partial remission before admission to hospital due to re-exacerbation). HC—healthy controls, UHR—patients with ultra-high risk of developing psychosis, FEP—patients with first episode psychosis, SCH—patients with chronic schizophrenia, BMI—body mass index, GAF—Global Assessment of Functioning, PANSS—Positive and Negative Syndrome Scale, SIPS—Structured Interview for Prodromal Syndromes, CRP—C-reactive protein, IL-6—interleukin-6, IL-8—interleukin-8, IL-10—interleukin-10, TNF-α—tumor necrosis factor-α, IFN-γ—interferon-γ.

**Table 2:** Spearman rank correlation coefficients (ρ) for inflammatory markers and fractional anisotropy (FA) of the left and right cingulum bundle (CB) in all analyzed groups.

	HC (*n* = 29)		UHR (*n* = 12)		FEP (*n* = 19)		SCH (*n* = 51)	
FA of CB	Left	Right	Left	Right	Left	Right	Left	Right
CRP	−0.190	−0.181	−0.014	−0.252	0.407	0.272	0.118	0.197
IL-6	−0.208	−0.410 *	−0.608 *	−0.462	−0.142	−0.281	−0.035	−0.088
IL-8	0.291	0.325	0.203	0.056	−0.304	−0.161	−0.030	−0.003
IL-10	0.175	0.178	−0.098	0.221	−0.283	−0.259	0.085	0.024
TNF-α	−0.197	0.008	0.014	0.154	−0.049	0.072	0.277 *	0.144
IFN-γ	−0.358	−0.398 *	0.079	0.248	−0.441	−0.159	0.061	0.104

* *p* < 0.05 (without correction for multiple comparisons, no statistically significant correlations after correction).

**Table 3:** Spearman rank correlation coefficients (ρ) for inflammatory markers and mean diffusivity (MD) of the left and right cingulum bundle (CB) in all analyzed groups.

	HC (*n* = 29)		UHR (*n* = 12)		FEP (*n* = 19)		SCH (*n* = 51)	
MD of CB	Left	Right	Left	Right	Left	Right	Left	Right
CRP	−0.085	−0.260	−0.098	−0.126	0.274	0.444	−0.009	0.066
IL-6	−0.090	−0.215	0.175	0.517	0.161	0.325	0.300 *	0.230
IL-8	−0.465*	−0.360	−0.594 *	−0.734 **	0.311	0.239	−0.025	−0.141
IL-10	0.300	0.197	0.130	0.098	0.004	−0.014	0.154	0.090
TNF-α	0.314	0.230	0.007	0.084	0.046	−0.054	0.091	0.205
IFN-γ	−0.123	−0.009	−0.370	−0.200	0.048	−0.396	0.214	0.184

* *p* < 0.05, ** *p* < 0.01 (without correction for multiple comparisons, no statistically significant correlations after correction).

## Data Availability

Data are available from the corresponding author on reasonable request.

## Supplementary Materials

The following supporting information can be downloaded at: https://www.mdpi.com/article/10.3390/jcm11216352/s1, Table S1: Analysis of associations between inflammatory markers and demographic, anthropometric and clinical parameters, and psychopathological symptoms.
